# Potential Role of HPA Axis and Sympathetic Nervous Responses in Depletion of B Cells Induced by H9N2 Avian Influenza Virus Infection

**DOI:** 10.1371/journal.pone.0051029

**Published:** 2012-12-10

**Authors:** Wenbao Qi, Jin Tian, Changhui Zhang, Jun He, Zhangyong Ning, Peirong Jiao, Ming Liao

**Affiliations:** 1 College of Veterinary Medicine, South China Agricultural University, Guangdong, People's Republic of China; 2 MOA Key Laboratory for Animal Vaccine Development, Guangzhou, People's Republic of China; 3 Key Lab of Zoonoses Control and Prevention of Guangdong, Guangdong, People's Republic of China; University College Dublin, Ireland

## Abstract

Except severe pulmonary disease caused by influenza virus infection, an impaired immune system is also a clinic characteristic. However, the mechanism(s) of influenza virus infection-induced depletion of B cells was unknown. Here, we compared the effect of two variant virulence H9N2 virus infections on mouse B cells. Our study found that the infection with highly pathogenic virus (V) of led to depletion of spleen B cells and bone marrow (BM) early B cells, compared to lowly pathogenic virus (Ts). Moreover, high apoptosis and cell cycle arrest in spleen and BM were detected, suggesting important factors for the reduction of B cells in both organs. Further, this effect was not caused by virus replication in spleen and BM. Compared to Ts virus infection, V virus resulted in higher glucocorticoids (GCs) and lower leptin level in plasma. Intraperitoneal GCs receptor antagonist RU486 injection was sufficient to prevent the loss of spleen B cell and BM pro- and immature B cells, but similar result was not observed in leptin-treated mice. Depletion of spleen B cells and BM pro-B cells was also reversed by chemical sympathectomy mediated by the norepinephrine (NE) analog 6-hydroxydopamine (6-OHDA), but the treatment didn't affect the GCs level. This study demonstrated that depletion of B cells induced by H9N2 AIV was dependent on HPA axis and sympathetic response.

## Introduction

Influenza A virus infection causes severe disease in humans and is an important topic in clinical health [1]. Severe destruction of lung tissue was observed by pathologists in human patients, which was further confirmed by mouse models of highly pathogenic avian influenza virus infection (HPAIV). Except severe pulmonary disease caused by influenza virus infection, an impaired immune system (e.g., elderly, infants, and immunosuppressed people), is mostly found in young and middle-aged people after HPAIV infection [2]. One hallmark of influenza virus infection in humans and other animal models was a strong reduction of T, B lymphocytes leading to limit the activation of immune responses [3]. The H5N1 virus (influenza A/Hong Kong/483/97 (HK/483)) infection led to significant decrease in the total number of circulating leukocytes [4]. But the thymus, spleen and lymphonode were targeted in the HK/483 of infection, so it could not conclude the lymphopenia was directly or indirectly mediated by the virus replication. So, the reason and mechanism for the declining number of lymphocytes remain unclear [4]. Recent studies using mice models to investigate the mechanism(s) of lymphopenia in severe influenza infection have implicated HPAIV infection was able to transport infectious virus from the lungs into the thymus causing functional damage of the thymus and interfering with T lymphocyte development, which was thought as a potential factor for T cells reduction [4].

A potential mechanism for the reduction of B cells in influenza infection remained less. Naïve mature B cells exported from the bone marrow (BM) experience a transitional phase in the spleen during which further maturation events lead to mature B cells production [5]. If aberrations occur during the process of B cell development, the perturbations to B cell homeostasis may ensue. While B cells response may play a minor role in early anti-virus immune response, excessive apoptosis may lead to immunodeficiency and impaired adaptive immune response.

Our previous studies had demonstrated that a H9N2 virus (A/chicken/Guangdong/V/2008 (V)) infection in mouse experiment could interfere with the development of T cells in thymus, similar to other studies (accepted by *PLoS ONE*). But the effect was caused indirectly by infection, because no virus was detected in lymphoid tissues. So, other pathways may contribute to the effect. Further, we found the glucocorticoids (GCs), a class of small lipophilic compounds whose receptor can be found in most immune cells, played a critical role in the loss of CD4^+^CD8^+^ thymocytes. It is necessary to investigate the role of GCs in the depletion of B cells in influenza virus infection.

It is well-demonstrated that the nervous and immune systems communicate through soluble mediators, such as cytokines, hormones, and neurotransmitters [6], [7]. Influenza A virus (IAV) and other viruses are known to activate both hypothalamic–pituitary axis (HPA) and sympathetic nervous system (SNS) [8]. The SNS, one arm of the autonomic nervous system, is responsible for the “fight or flight” response. Studies from Kristie M. Grebe et al. have proved that IAV-specific CD4 and CD8 T cell responses was suppressed by A/Puerto Rico/8/34 (PR8) and 6-OHDA-mediated ablation of the mouse peripheral sympathetic nervous system increases primary CD8 T cell responses [9]. Another reports from them further demonstrated that peripheral sympathectomy reduces mouse morbidity and mortality from PR8-infected mice due to reduced inflammatory influx of monocytes, neutrophils, and NK cells [10]. It is necessary to investigate the role of sympathetic nervous response in the depletion of B cells in influenza virus infection.

In this study, we systematically investigated the effect of variant virulence H9N2 AIV on early and mature B cells respectively from central and peripheral immune organs and found the infection with highly pathgenetic H9N2 AIV induced a reduction of spleen B cells and BM early B cells. Further, higher GCs level and activated sympathetic nervous response were identified as potential mechanisms for the loss of B cells. Moreover, the higher GCs production was independent of the activated sympathetic nervous system. This conclusion firstly provided insight into the role of nervous and endocrine system in the loss of B cells during H9N2 influenza virus infection.

## Materials and Methods

### Viruses

The H9N2 AIVs used in this study were: A/chicken/Guangdong/TS/2004 (TS) and A/chicken/Guangdong/V/2008 (V). The V virus infection displayed highly pathogenic to mice whose infection was lethal, and Ts virus displayed lowly pathogenic to mice whose infection was non-lethal (another studies accepted by *PLoS ONE*). The viral titer was determined by plaque assay on MDCK cells (ATCC) in duplicate.

### Mice Infections

Six-week old female BALB/c mice (n = 12/group) (Experimental Animal Centre of Guangdong Province) were anesthetized with dry ice and intranasally (i.n.) inoculated with 50 µl of 10^4^ PFU of influenza virus. At indicated time point post-infection, mice (n = 3/group) were euthanatized and the blood, spleen, right femur, and mediastinal lymph node (MLN) were collected for analysis. All animal research was conducted under the guidance of CDC's Institutional Animal Care and Use Committee and in an Association for Assessment and Accreditation of Laboratory Animal Care International-accredited facility. The animal research in our study had been approved by Guangdong Province Animal Disease Control Center.

### Protocols for intraperitoneal injection experiments

Mice were divided into four groups (n = 6/group). All the groups were allowed ad libitum access to water and food. One group was allowed to receive an intraperitoneal injection of 0.1 mg/g (initial weight) of mifepristone (RU486, an specific inhibitor of GCs receptor) (Sigma-Aldrich) (suspended in 100 µL of 2% ethyl alcohol) daily; the second group was allowed to receive an intraperitoneal injection of 2 ug/g (initial body weight) of leptin (ProSpec) daily; the third group was allowed to receive an intraperitoneal injection of both hormones (same dose referred above) daily; the mock group was allowed to receive an intraperitoneal injection of 100 µL of 2% ethyl alcohol. All the injections started one day before infection and was kept until the end of the experiments. At the same time, to check the effect of GCs and leptin on B cells, another four mock groups were established without virus infection.

### Chemical sympathectomy of mice

A classic model for investigating the influence of the SNS on host immune responses is based on chemical sympathectomy mediated by the NE analog 6-OHDA. 6-OHDA is selectively transported via NE receptors into peripheral sympathetic nerve termini, where it is oxidized to generate free radicals that destroy the termini [10]. Nerve regeneration needs at least a month, providing a chance to examine host immune response in the absence of a functional peripheral SNS. Mice (n = 6/group) were treated with 200 mg/kg 6-hydroydopamice (6-OHDA) (Sigma-Aldrich) in 0.9% Nacl plus 10^−7^ M ascorbic acid (Sigma-Aldrich) on day-3 and 100 mg/kg on day-5 and day-7. Control mice received injections of 0.9% Nacl plus 10^−7^ M ascorbic acid. Sympathectomy was confirmed in the spleen frozen sections by sucrose-phosphateglyoxylic acid reaction as described [10]. At the same time, to check the effect of 6-OHDA on B cells, another two mock groups were established without virus infection.

### Flow cytometric analysis for B cells

The following mAbs for flow cytometry were used: anti-CD43, anti-IgD, anti-IgM and anti-B220 (BD Bioscience). The peripheral blood, spleen, MLN and right femur from three mice per group per time point were collected. Spleen and MLN were gently passed through a 200-micron nylon mesh, lysed with NH_4_Cl-Tris buffer, and single cell suspensions were washed and resuspended in PBS. BM cells were flushed from the right femur by using PBS and lysed with NH_4_Cl-Tris buffer. Next, 0.1 ml of blood or single cell suspensions containing 10^6^ cells was incubated on ice for 10 min with anti-Fc block (anti-CD16/32). Specific cell populations were stained with anti-CD45/B220 for analysis of total B220^+^ B cells and anti-CD45/B220, anti-CD43, anti-IgD and anti-IgM for analysis of B cells in BM. Following staining for 30 min, the erythrocytes in blood samples were lysed with Optiman C (Beckman), and the samples were added with 1 ml PBS and analyzed on FACSCalibur flow cytometer (BD Bioscience). Other samples following staining for 30 min were washed twice, resuspended in 1 ml of 2% paraformaldehyde, and analyzed on FACSCalibur flow cytometer. B lineage cells in BM were distinguished by expression of cell-specific markers as indicated in reference [11], [12]. Briefly, a total of 10,000 events gated for leukocyte-gated (residual erythrocytes only excluded) cells (Gate R1) were selected by FSC/SSC; secondly, the B220/FSC was used to gate for total B220^+^ cells (Gate R2); thirdly, the B220/IgM were used to gate for pre/pro- B cells (B220^+^IgM^−/low^, Gate AX), immature B cells (B220^+^IgM^+^,Gate AW) and mature naïve B cells (B220^high^IgM^+^, Gate AV) and finally, the Gate AX were further analyzed basing on IgD/IgM to identify the pro-B cells (B220^+^CD43^high^, Gate F) and pre-B cells (B220^+^CD43^low/−^, Gate G). The proportions of BM B lineage cells against total B220^+^ cells and total BM cells were calculated. The numbers of viable cells per sample was determined by using a Coulter counter (Beckman), and individual cell subsets were calculated by multiplying the percentage of each cell type (as determined by FACS) by the total number of viable cells per tissue.

### Analysis of apoptosis and cell cycle

Mice (n = 5/group) were infected with each virus (10^4^ PFU). At day 5 post infection, spleens and BM from each group were collected. The single cell suspensions from three spleens were prepared and 0.1 ml of cell suspensions containing 10^6^ cells was stained with annexin-V, PI (Invitrogen) and anti-B220 according to the manufacturer's instructions. Following stained, the cells were immediately analyzed on a FACSCalibur flow cytometer. A total of 10,000 events gated for B220^+^ B cells were performed in three independent experiments. Positioning of quadrants on annexin-V and pi dot plots was performed. Late apoptotic cells with DNA strand breaks were identified in histological paraffin sections using the in situ terminal deoxynucleotidyl transferase-mediated dUTP-biotin nick end labeling (TUNEL) kit (Sigma-Aldrich). A total of six paraffin sections from two spleens per group were prepared according to the manufacturer's instructions. Three independent experiments were performed. The brown cells were TUNEL-positive cells. Cell cycle analysis was performed using propidium iodide (Sigma-Aldrich) as described [13]. Briefly, 0.1 ml of cell suspensions containing 10^6^ cells was suspended in staining buffer (including 100 µg/ml RNase A) and stained with annexin-V, PI (Invitrogen) and anti-B220 according to the manufacturer's instructions. In this case, 10,000 events gated for B220^+^ B cells were collected, and histograms were analyzed using MultiCycle AV software.

### Quantitative Real-time PCR

Mice (n = 12/group) were inoculated i.n. with 10^4^ PFU of V and Ts. The spleens and BM cells per group per time point were harvested from day 1 to 7. Total RNA was isolated from the homogenate using TRIzol reagent (Invitrogen). The allantoic fluids from eggs inoculated with virus and lungs from infected mice were worked as the positive control. The cDNA was achieved with reverse-transcibed Kit (Promega). Quantitative real-time PCR was used to determine the expression of beta-actin and influenza virus M gene with SYBR Green PCR Kit (TAKARA). The relative expression values of M gene were normalized to the expression value of the β-actin gene. The qPCR programs and primer sequences could be supplied if needed.

### Cortisol and leptin quantitation

Mice (n = 12/group) were infected with 10^4^ PFU of each virus. Peripheral blood samples were collected from the orbital plexus of anaesthetic mice (n = 3/group/time point) and the plasma was prepared. Plasma levels of cortisol (Enzo) (assay sensitivity, 50 pg/ml) and leptin (Millipore) (assay sensitivity, 50 pg/ml) were measured by ELISA.

### Statistical analysis

Statistical significance of differences between experimental groups was determined through the use of unpaired t test and ANOVA with Prism 5.0 software (GraphPad Software). *p*<0.05 was thought significant difference.

## Results

### The infection with V virus leads to depletion of B cells in peripheral lymphoid tissues and blood

**Figure 1:**
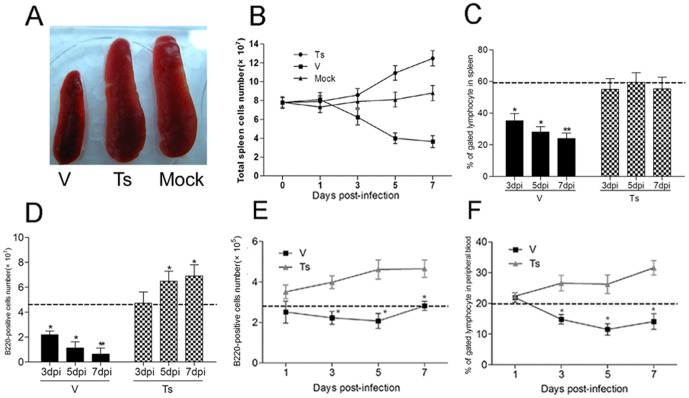
The infection with V led to the atrophy of spleen and the reduced B220^+^ B cells in spleen, MLN and peripheral blood. Following infection with V and Ts, the spleen (A), MLN and blood from three mice per group per time point were collected, and the numbers of spleen cells (B) were calculated the percent of B220^+^ B cells were analyzed by flow cytometry. The proportion (C) and amount (D) of B220^+^ B cells in spleen were analyzed on day 3, 5 and 7 post-infection. The amount of B220^+^ B cells in MLN (E) and proportion in peripheral blood; (F) were analyzed on day 1, 3, 5 and 7 post-infection. Baseline from PBS inoculated mice (n = 5) is shown as a dashed line in each graph. The numbers of B220^+^ B cells in spleen and MLN were calculated by multiplying the percentage of each cell type by the total number of their viable cells. The data shown represents mean ± SD for three independent experiments. * *p*<0.05 and ** *p*<0.01 between V and Ts.

During infection with V virus, the spleen underwent a strong atrophic process that led to a drastically reduced size at day 7 p.i., which was not observed in the Ts-infected group (Fig. 1A). Moreover, the total splenocyte amount was drastically reduced with 3.4 times compared with that in uninfected mice, but increased with 1.4 times in Ts-infected mice at day 7 (Fig. 1B).

One significant characteristic from experimental infection of chickens and mice with the avian influenza virus is the destruction of lymphocytes [3]. So, it was important to check whether the atrophy of spleen would affect the numbers of B cells in spleens as well as other peripheral lymphoid tissues. Following challenged with V and Ts virus (10^4^ PFU), mice were anesthetized and spleen, MLN and peripheral blood were collected for analysis of B220^+^ B cells at day 1, 3, 5 and 7. A progressive loss of B220^+^ B cells was observed in the spleen of V-infected mice 3–7 days p.i., but not in Ts-infected mice (Fig. 1C and D). In comparison with Ts group, the proportion of B220^+^ B cells in V group was reduced ∼35% (* *p*<0.05) and the amount of B220^+^ B cells was reduced nearly 2-fold (* *p*<0.01) on day 3. And the significantly lower levels of B220^+^ B cells from the spleens of V-infected mice were also detected at day 5 and 7. In the MLN and periphery blood from V-infected mice, the reduction of B220^+^ B cells was also observed (Fig. 1E and F) from day 3 to 7, compared to Ts-infected mice. So the infection with virulent H9N2 virus led to depletion of B cells in peripheral lymphoid tissues and blood.

### The depletion of B cells in spleen is due to the increased apoptosis and cell cycle arrest

**Figure 2:**
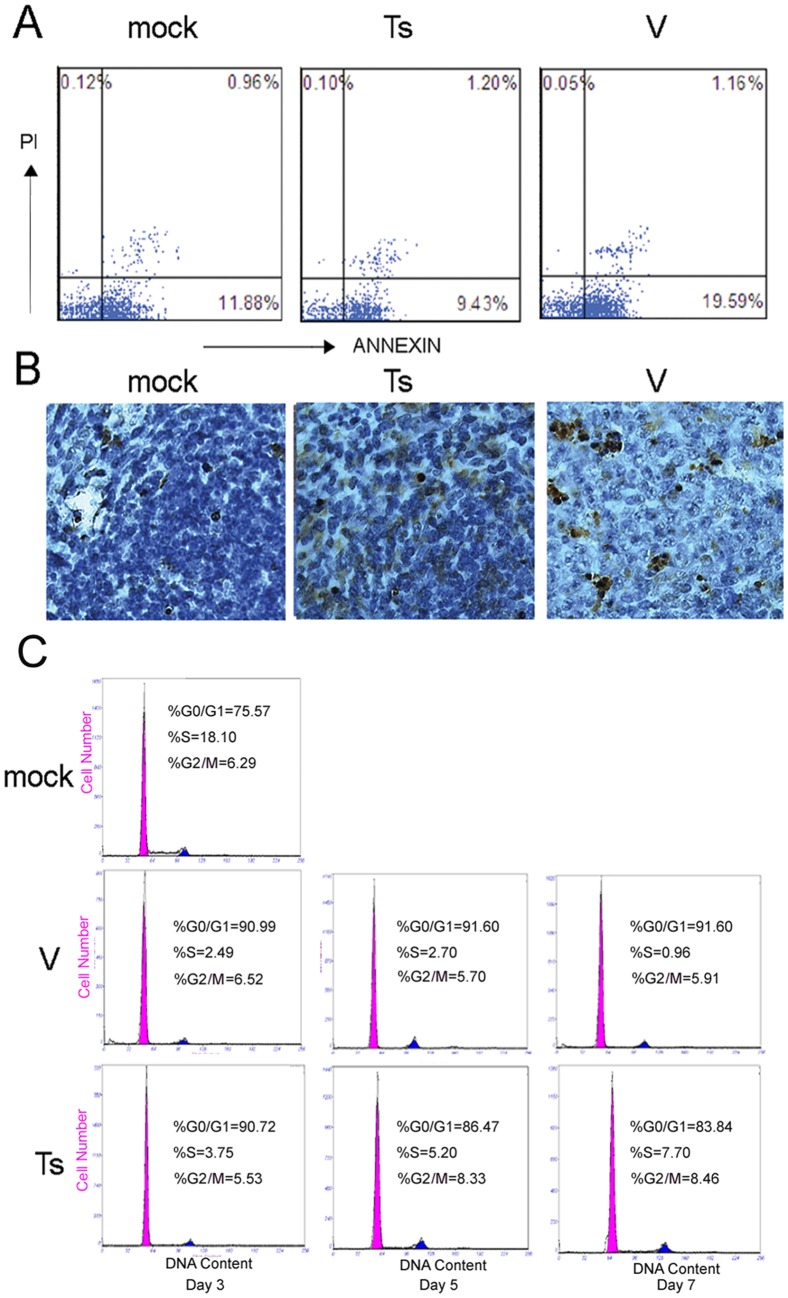
The depletion of B220^+^ B cells in spleen is due to the increased apoptosis and arrested cell cycle. (A) Splenic cells from mice 5 days after infection with V and Ts were stained with PE-conjugated anti-B220 mAb, annexin V and propidium iodide and analyzed by flow cytometer. Numbers indicate the percentages of early apoptosis cells (Annexin-V^+^) and late apoptosis cells (Annexin-V^+^PI^+^) within B220 gated cells populations. The number was estimated by collecting 10,000 events. (B) After challenging, mice (n = 3/group) were euthanized on 5 days p.i. and the histological paraffin sections of spleens were prepared. Apoptotic cells in histological spleen sections were identified using the TUNEL assay. Magnification, ×200. (C) Following infection, spleen cells were prepared and stained with PE-Cy5-anti-FITC and PI and analyzed by flow cytometer. The results were analyzed using MultiCycle AV software. Each graph is from an individual mouse, but is representative of three mice per group and three independent experiments.

Newly formed B cells emigrate from the bone marrow and home initially to the spleen where they mature via transitional stages mainly into long-lived follicular B cells and marginal zone (MZ) B cells [14]. So, the reduction of B cells in circulating blood and MLN may be resulted from the loss of spleen B cells. Next we examined the factors for the loss of spleen B cells. Flow cytometry and TUNEL assay for apoptotic analysis were performed following infection with V and Ts (10^4^ PFU). At day 5, the spleens from three mice per group were removed and single cells suspensions were prepared. The apoptosis percent of B220^+^ B cells at the early stage of apoptosis after V infection had a significant increase (18.47±3.81%) compared with Ts-infected group (7.47±1.73%) (* *p*<0.05) (Fig. 2A). Situ detection of cells with DNA strand breaks in spleen sections was analyzed using TUNEL assay on day 5. In the mock and Ts infected mice, only a few TUNEL-positive cells could be observed, but more in the V-infected mice (Fig. 2B).

To check whether cell cycle arrest was associated with the reduction of B220^+^ B cells, we assessed the cell cycle of B220^+^ B cells at day 3, 5 and 7 (Fig. 2C). The infection with V and Ts (10^4^ PFU) both contributed to increased proportion of cells in G0/G1 phase and decreased proportion of cells in S and G2/M phase at day 3 and 5, compared to mock group. But, there was no significant difference between both groups. On day 7, the proportion of cells in G0/G1 phase of cell cycle in V-infected group increased to 90.47±3.82% and the proportion in Ts-infected group recovered to 80.22±2.52%, which showed that the progression of B cells into S phase of the cell cycle is blocked in V-infected mice. The comparisons of cell cycles between V and Ts groups revealed that the significant difference did not coincide precisely with the initial detectable loss of spleen B cells, so it may play a minor role in the increased apoptosis and depletion of B cells in V-infected mice.

### V virus infection leads to depletion of pro-, pre- and immature B cells in BM

**Figure 3:**
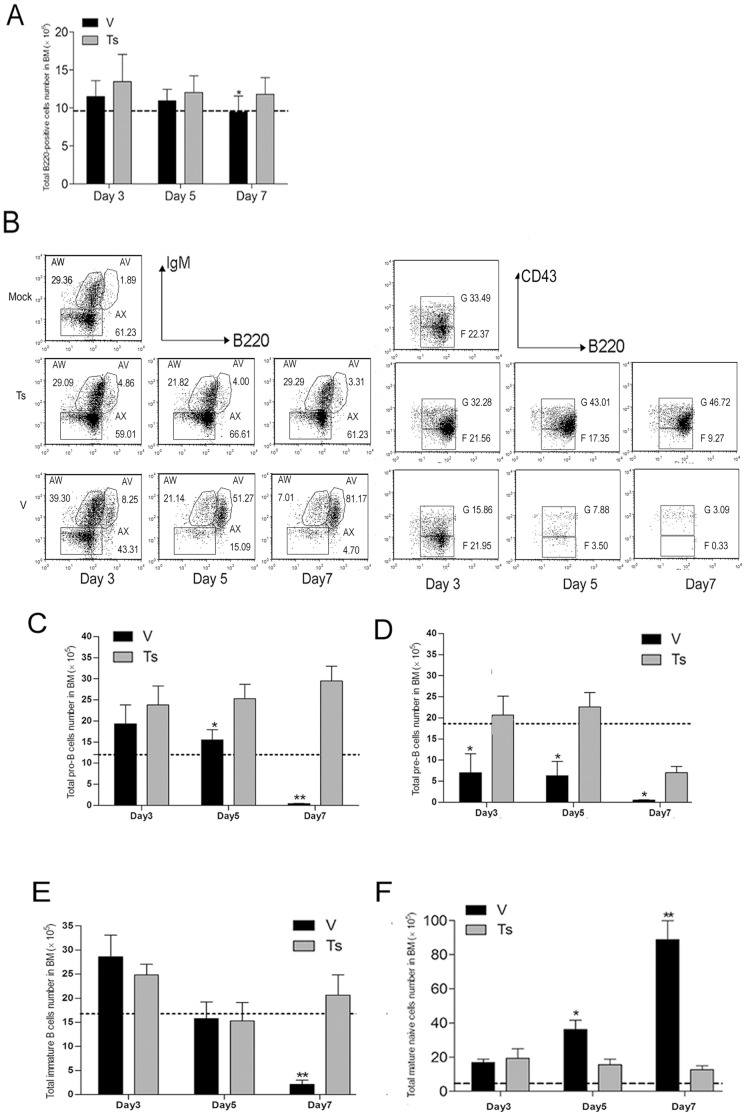
Alternation of BM B cells populations following virus infection. Following infected with V and Ts virus, the BM cells from three mice per group per time point was prepared on day 3, 5 and 7 post-infection and the phenotypic FACS analysis of B220, IgM, IgD, CD43 expressions within BM cells was performed. (A) The numbers of B220^+^ cells within BM; (B) the percentages of BM B lineage cells within B220 gated cells populations. B220^low^IgM^−^ cells define pro-B (B220^+^CD43^high^) and pre-B (B220^+^CD43^low/−^) cells, B220^low^IgM^+^cells are immature B cells and B220^high^IgM^+^ cells are mainly mature naive recirculating B cells. Percentages shown are calculated from total B220^+^ cells (erythrocyte-depleted). The numbers of pro- (C), pre- (D), immature B cells (E) and mature naïve B cells (F) were calculated by multiplying the percentage of each cell type by the total number of BM viable cells. Data shown in graph A, C, D, E, F represents mean ± SD for three independent experiments. Data shown in graph B are two-color dot plots from individual mouse, but are representative of three independent experiments. * *p*<0.05 and ** *p*<0.01 between V and Ts.

To further study whether the influenza virus infection would affect the B lineage cells in BM, we characterized the alterations in BM B lymphocytes during infection i.n. with V and Ts (10^4^ PFU) (Fig. 3). After infection, three mice per group were anesthetized at day 3, 5, 7 p.i. and the right femurs were harvested. BM cells were flushed by using PBS and were analyzed by flow cytometry following staining. Compared with Ts-infected mice, the numbers of total B220^+^ B cells in BM of V-infected mice exhibited a reduction only at day 7 (Fig. 3A). The ratios of pro-/pre-B cells in total B220^+^ B cells exhibited a progressive reduction compared with Ts-infected mice from day 3 to 7 (Fig. 3B). The numbers of pro-B cells in V-infected mice were significantly lower (* *p*<0.05) than those in Ts-infected mice at day 5 and 7 (Fig. 3C), and similarly lower level for the pre-B cells in V-infected mice was detected from day 3 to 7 (Fig. 3D). The percentages and numbers of immature B cells in V-infected mice were significantly reduced (* *p*<0.01) at day 7, compared with Ts-infected mice (Fig. 3B, 3E). A decrease of nearly ten-fold immature B cells (2.1±0.16E10^5^) was observed in V-infected mice compared to the number (2.0±0.16E10^6^) in Ts-infected mice. B220^high^IgM^+^IgD^+^ cells in the BM are mature naïve recirculating B cells. Studies from Annaiah Cariappa have demonstrated that BM may provide an alternative niche for IgD^hi^ B cells and these cells could generate immune response to blood-borne pathogens [15]. The ratios and numbers of mature naïve B cells in V and Ts group were both increased compared with mock group (Fig. 3B, 3F). At day 7, the proportion of this population in B220^high^ B cells reached >90% in V-infected group, but was kept at 10%-15% in Ts-infected mice (Fig. 3F). These data indicated that V virus infection induced a dramatic reduction of pro-, pre- and immature B cells in BM and interfered with B cells development, which may be one of important factors for depletion of B cells in peripheral organs.

### BM B cell depletion is due to cell cycle arrest and apoptosis

**Figure 4:**
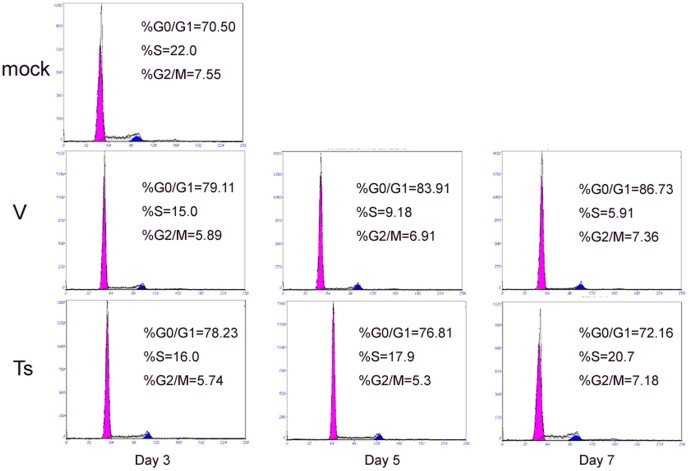
The depletion of B220^+^ B cells in BM is associated with arrested cell cycle. Following infection, BM cells from day 1, 3, 5, 7 post-infection were prepared and stained with PE-Cy5-anti-FITC and PI and analyzed by flow cytometer. The results were analyzed using MultiCycle AV software. Each graph is from an individual mouse, but is representative of three mice per group and three independent experiments.

It was possible that the loss of BM early B cells was caused by cell cycle arrest followed by cell death within the BM [13]. Therefore, the cycling characteristics of BM B220^+^ cell population (exclude B220^high^ cells) were assessed during the course infection. The proportions of B220^+^ cells (pro-B, pre-B, and immature B cells) in the S phase of cell cycle from V- and Ts-infected mice were decreased, but there was no difference between virus-infected mice and PBS-infected mice at day 3 (Fig. 4). However, at day 5 and 7, the proportions of B220^+^ cells in the S phase of the cell cycle from V-infected mice were significantly lower than Ts-infected and mock mice. Thus the progression of early B cells into S phase of the cell cycle is blocked in V-infected mice. The experiment of apoptosis showed that the apoptosis percent of B220^+^ B cells (exclude B220^high^ cells) at the early stage of apoptosis after V infection had a significant increase compared with Ts-infected group (*p<0.05) at day 5 and 7 (data not shown). This coincided precisely with the dramatic loss of BM B cells at day 7. So, the reduction of BM early cells may be associated with the inhibition of G1/S transition and increased apoptosis.

### The depletion of B cells in spleen and BM depends on GCs but not leptin

We subsequently examined the major mechanism responsible for loss of B cells after V infection. HPAIV H5N1 and H7N7 could reach the thymus by abusing the homing process of DCs of lung, which interfered with the T lymphocyte selection process and led to the reduced outflow of mature T cells [4]. So we examined the level of virus nucleic acids in spleen of V-infected mice by qPCR, but could not detect the virus nucleic acids during the course of infection (data not shown).

**Figure 5:**
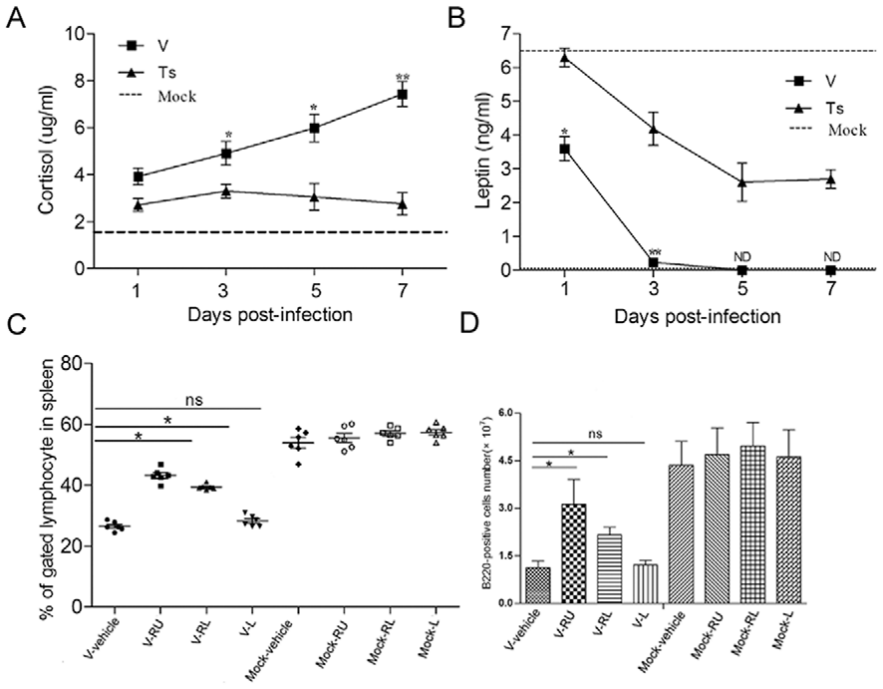
Role of GCs and leptin in depletion of spleen B cells. (A–B) Following infected with V and Ts virus, plasma from mice was prepared at different time points. Cortisol and leptin levels in plasma were measured using ELISA. Baseline from PBS inoculated mice (n = 5) is shown as a dashed line in each graph. ND means the examination is lower than the limit of detection. Data represent means of three mice per group per time point ± SD. *p<0.05 and **p<0.01 between V and Ts. (C–D) The mice were divided for four groups (12 mice/group) and inoculated i.p. respectively with 2% ethyl alcohol (vehicle), RU486 (R), RU486 plus leptin (RL), leptin (L) from day −1 p.i. and until the end of experiment. Each group was divided for two groups (6 mice/group) again, and one group was infected i.n. with 10^4^ PFU of V (V-) and another was infected i.n. with PBS (Mock-). The spleen cells were prepared on day 5 post-infection and stained. The percentages (C) each group were evaluated by flow cytometry, and the numbers (D) were calculated by multiplying the percentage of each cell type by the total number of spleen viable cells. Data shown in C and D represent means of 6 mice from each group ± SD. Ns mean no statistical difference between both. *p<0.05 and **p<0.01 between V-vehicle and V-R or V-RL or V-L.

Glucocorticoids (GCs) are a class of steroid hormones that display potent immunomodulatory activities including the ability to induce lymphocyte apoptosis [16], [17], [18] and leptin plays an important role in promoting B cells survival and sustaining its homeostasis [19]. So we examined the levels of both hormones during the V and Ts infection. The results showed that both infections increased the production of cortisol and decreased the production of leptin in the plasma compared with mock group, but the altered magnitude between both infection groups was variant (Fig. 5A, 5B). The levels of cortisol in V-infected mice were significantly higher (*p<0.05) than those in Ts-infected mice from day 3 to 7. The levels of leptin in V-infected mice were significantly lower than those in Ts-infected mice (*p<0.05) at each time point measured. So, to check whether the alternation of both hormones level was responsible for the reduction of B cells in spleen and BM, mice (n = 3/group) were administrated i.p. with RU486 (V-R) or leptin (V-L) or RU486 plus leptin (V-RL), respectively. Following challenging with 10^4^ PFU of V virus, single cells suspensions from spleen and BM were prepared at day 5. The early apoptosis ratio of spleen and BM B220^+^ B cells from group V-R and V-RL was reduced, but no significant change in group V-L compared to the V-alone group (data not shown). As an important note, the percentages and numbers of B220^+^ B cells in spleen in group V-R or V-RL were significantly increased, but the protected effect of administration with RU486 alone was higher than the administration with RU486 and leptin together (Fig. 5C, 5D). But the administration with leptin alone (recover to the normal level) did not rescue the loss of total B cells. As shown in Table 1, the administration with RU486 mainly increased the numbers of pro-B, immature B cells and total B220^+^ B cells in BM, and decreased the number of mature naïve B cells, compared with V-alone group. All these data showed that higher GCs levels were responsible for the depletion of B cells in spleen and BM.

**Table 1:** Numbers of B lineage cells in BM following treatment with RU486 and/or Leptin.

Groups	Numbers of BM B cells populations^a^				Numbers of B220^+^ B cells (×10^6^)
	Pro-B (×10^6^)	Pre-B (×10^6^)	Immature B (×10^6^)	Mature B (×10^6^)	
V-R	5.30±0.12^b^	1.29±0.14	4.88±0.51^b^	1.89±0.21^b^	13.78±1.11^b^
V-RL	3.71±0.11	1.10±0.13	3.72±0.45	3.15±0.45	12.34±1.45
V+L	2.65±0.21	0.95±0.08	1.75±0.21	5.32±0.53	10.75±1.34
V-vehicle	2.79±0.13	1.15±0.09	2.89±0.22	3.64±0.56	11.37±1.28
Mock-R	3.71±0.19	2.72±0.42	3.19±0.33	0.82±0.05	9.87±1.03
Mock-RL	3.66±0.22	2.80±0.38	2.89±0.25	0.87±0.06	9.67±1.42
Mock-L	4.07±0.36	3.04±0.41	3.19±0.38	0.75±0.05	10.28±1.55
Mock-vehicle	4.97±0.32	3.01±0.35	3.29±0.39	0.79±0.04	10.23±1.68

a Following treatment with RU486 (R), RU486 plus leptin (RL), leptin (L), vehicle respectively, mice were infected i.n. with 10^4^ PFU of V (V-) or PBS (Mock-), respectively. The right femurs from six mice per group were collected at day 5. BM cells were prepared and stained with anti-CD45/B220, anti-CD43, anti-IgD and anti-IgM, and analyzed by flow cytometer. The numbers of different B cells populations were calculated by multiplying the percentage of each cell type by the total number of BM viable cells. Data is representative of means of six mice ± SD for three independent experiments..

b Indicates statistically significant differences (*p*<0.05) comparing to data in V-vehicle group.

### Sympathetic nervous system directly participates in the depletion of B cells during the V infection, which is not mediated by higher GCs level

**Figure 6:**
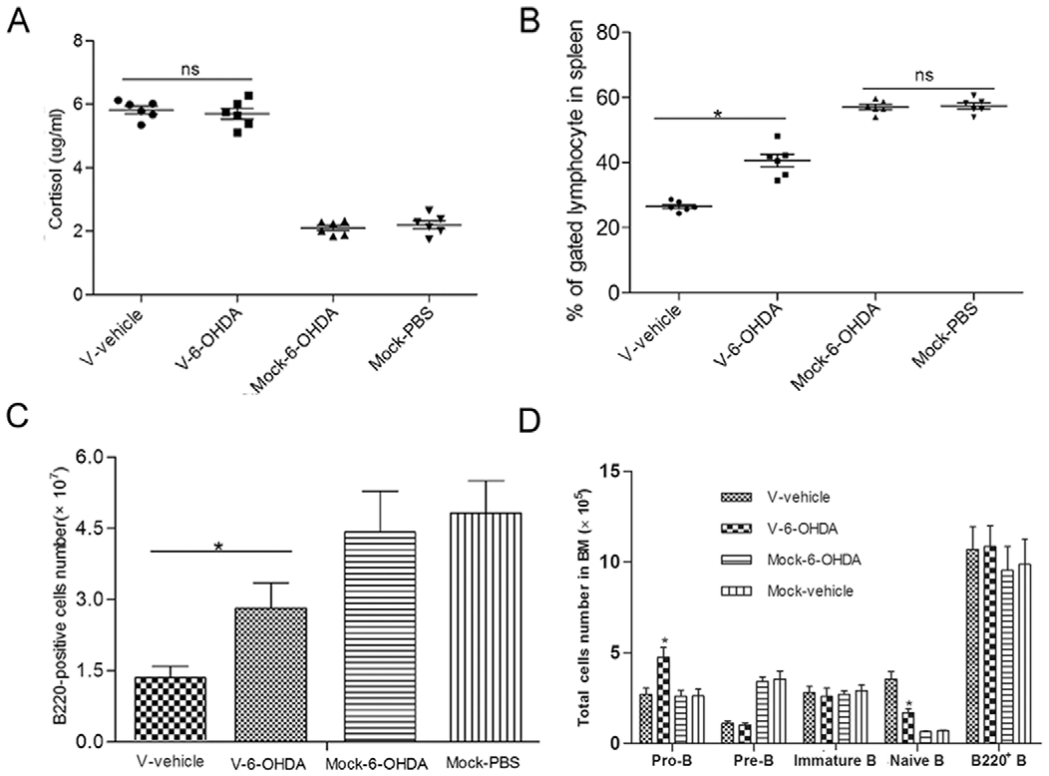
Role of sympathetic nervous system in the production of GCs and depletion of spleen and BM B cells. Following 6-OHDA treatment, six mice were infected i.n. with 10^4^ PFU of V (V-6-OHDA) and another six mice were infected i.n. with PBS (mcok-6-OHDA). Another two groups (6 mice/group) treated with vehicle were infected i.n. with 10^4^ PFU of V (V-alone) or PBS (Mock-vehicle), respectively. (A) GC levels in plasma of each group were measured using ELISA. After infected with V virus, spleen and BM cells were prepared on day 5 post-infection. The proportions (B) and numbers (C) of splenic B220^+^ B cells were determined. The numbers of pro-, pre-, immature B cells and mature naïve B cells from BM (D) were calculated by multiplying the percentage by number of total BM cells. Data bars represent means of 6 mice from each infection group ± SD. * *p*<0.05 and ** *p*<0.01 between V-vehicle and V-6-OHDA. Ns mean no statistical difference between both.

The HPA axis could be activated by stressor-induced hypophysiotrophic neurons resulting in the synthesis and release of glucocorticoid hormones. But the adrenal cortex can also be directly activated by the sympathetic nervous system, which can regulate corticosteroid release [20]. To better understand whether the higher GCs level depended on the sympathetic nervous system (SNS) activation, we treated mice with 6-OHDA three times over a one-week interval. The 6-OHDA treated mice after infected by V showed an identical level of GCs with mock mice (Fig. 6A), which showed that the higher GCs production was independent of the sympathetic nervous system. The data revealed that the V infection as a stronger stressor directly activated the HPA axis and resulted in more GCs production. Similar to the effect of RU486, 6-OHDA treatment reduced the loss of spleen B cells after V virus infection. Compared with V-alone infection group (26.46±0.8%), the B cells in the spleen of 6-OHDA treated mice was increased to 40.16±3.5% (Fig. 6B). The amount of B220^+^ B cells was increased to 2.9E10^7^ from 1.4E10^7^ in V-alone infection group (Fig. 6C). As shown in Fig. 5C, 6-OHDA treatment significantly increased the pro-B cells and reduced the mature naïve B cells in BM, but did not affect the immature B cells and total B220^+^ cells, which was different from the effect of RU486 treatment.

These results suggested that higher GCs production was determined only by HPA axis, but the depletion of B cells in spleen and BM was associated with both HPA axis and sympathetic nervous response.

### Comparison of spleen and BM B cells from individual and both pathways blockade mice

**Figure 7:**
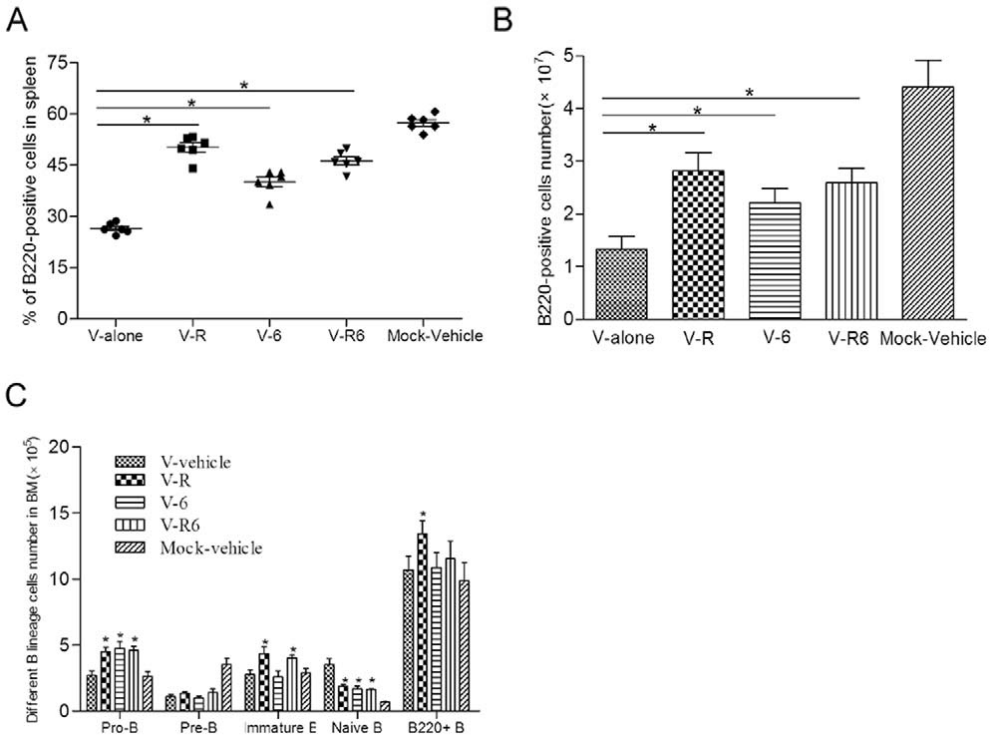
Comparison of spleen and BM B cells from individual and both pathways blockade mice. For this experiment, five groups mice (n = 6/group) were prepared. One group (V-6) mice was administrated with 6-OHDA before infection; Second group (V-R) was administrated with RU486 daily during infection; Third group (V-R6) was administrated with 6-OHDA before infection and RU486 daily during infection; Forth group (V-vehicle) was administrated with vehicle (2% ethyl alcohol ethyl alcohol). All the four groups were received with 104 PFU of V virus. The fifth group (Mock-vehicle) was administrated with vehicle and received with PBS as mock group. At day 5 post-infection, spleen and BM cells were prepared. The proportions (A) and numbers (B) of splenic B220^+^ B cells were determined. The numbers of pro-, pre-, immature B cells and mature naïve B cells from BM (C) were calculated by multiplying the percentage by number of total BM cells. Data bars represent means ± SD. * *p*<0.05 and ** *p*<0.01 between V-vehicle and V-R/V-6/V-R6.

To compare the effect of individual and both pathways blockade, we treated mice with 6-OHDA three times over a one-week interval and/or RU486 daily during infection. Following challenging with 10^4^ PFU of V virus, single cells suspensions from spleen and BM were prepared at day 5. Both HPA axis and SNS response or individual blockade raised the proportion and numbers of B220^+^ B cells in spleen compared with that in V-vehicle treated mice (Fig. 7A, 7B). But, both pathways blocking did not result in an overlapping effect. The spleen B220^+^ B cells in both blockade mice were more than that in SNS treated mice, and lower than that in RU486 treated mice. The similar results also happened in BM B lineage cells. Comparing with those in V-vehicle treated mice, both pathways blocking increased the numbers of Pre-, Immature B cells and decreased the naive B cells, the change of which was similar to the results with RU486 administration (Fig. 7C). But it did not increase the amount of BM total B220^+^ B cells, the change of which was similar to the result with 6-OHDA administration.

## Discussion

The data presented in this report firstly provides evidence that the HPA axis and sympathetic nervous system are involved in the loss of spleen B cells and BM early B cells caused by H9N2 AVI. We observed that the V virus infection led to the acute atrophy of spleen and B lymphopenia in peripheral tissues and BM. The depletion of B cells mainly resulted from the increased apoptosis and arrested cell cycle. Further, our results demonstrated that RU486 and 6-OHDA treatment both could rescue the loss of B cells in spleen and BM.

Leukopenia has been demonstrated following infection with a lot of viruses, and a transient leukopenia in humans could occur following infection with influenza virus [3], [4]. Highly lethal H5N1 influenza virus can induce severe systemic lymphoid depletion in experimentally infected mice, chicken and infected patient, which suggested some insight into the mechanism(s) of pathogenicity of influenza virus. So it was important to investigate the mechanism(s) responsible for depletion of lymphocytes. However, lethal H5N1 influenza virus could infect immune organs (e.g. thymus, spleen and lymphnode), so it is difficult to decide that the reduction of lymphocytes was caused by virus, directly or indirectly. With the rapid evolution of H9N2 AIV, a new virus isolate that is highly pathogenic to mice but with no replication in lymphoid organs provided a good research model for understanding the lymphopenia induced by influenza virus.

The infection with HKx31 (H3N2) strain of influenza A virus could cause a severe loss of BM pre- and immature B cells, which resulted mainly from cell cycle arrest and apoptosis within the BM environment, rather than from increased trafficking of BM emigrants to peripheral lymphoid tissues [12]. Different from that, in our study, all three types of early BM B cells were reduced following V infection. Naïve B cells in peripheral tissues are from BM immature B cells. The fact that there was a concomitant decrease in B220^+^cells in the peripheral tissues, is consistent with the reduction of B220^+^IgM^high^ BM B cells (Fig. 1), which further indicates that BM early B cell loss most likely exacerbates B cell loss within spleen and other tissues.

Obvious characteristics in influenza virus (HKx31) infection were cell cycle arrest and high apoptosis within BM, suggesting that both played an important role in influenza virus-induced reduction of early BM lineage cells [12]. The G1/S of transition is blocked in influenza virus-infected mice and most lymphocytes undergoing apoptosis in vivo are detectable. The alternation of cell cycle and apoptosis is dependent on TNF-α, lymphotoxin-α, and both TNF receptors. TNF-α^−^/^−^, LT-α^−^/^−^, and TNF-α^−^/^−^LT-α^−^/^−^ mice lead to resistant to BM pre-B/immature B cell depletion after influenza virus infection [12]. Similar to that study, arrested cell cycle and increased apoptosis was found in V infection group. Next, we would investigate whether H9N2 AIV-induced depletion of B cells was associated with TNF-α, lymphotoxin-α, and both receptors.

Within the immune system, apoptosis is a central mechanism for the normal lymphocyte homeostasis both during early lymphocyte development and in response to antigenic stimuli [21], [22]. B lymphocytes undergo apoptosis in many instances in their development, homeostasis, and activation. Studies from Queenie Lai Kwan Lam demonstrate that leptin promotes B-cell survival by inhibiting apoptosis and by inducing cell cycle entry through the activation of expressions of B-cell CLL/lymphoma 2 (Bcl-2) and cyclin D1 [19]. Because of the important role of leptin in sustaining B cells development and homeostasis in spleen and BM [23]–[26], we investigated the level of leptin in plasma. Lower leptin level even absent was observed in V infection group, and it may be expected to contribute to the effect in virus infection. However, increasing the leptin level of V-infected mice failed to rescue the loss of B cells, which decreased the survival rate (data not shown), instead. So, leptin did not play a critical role in V-induced depletion of B cells.

GCs have long been used as anti-inflammatory agents and anticancer treatments [27]. In our previous study, the reduction of inflammatory cells in V-infected lungs was observed due to higher GCs level, which inhibited anti-virus immune response. And, GCs are known to lead to G1 cell cycle arrest in human leukemic T cells [28] and transformed lymphoid cell lines [29]. In our study, arrested cells cycle in B220+ B cells of spleen and BM was presented revealing the critical role of GCs in this. While many genes affected by GC treatment are critical for progression of the cell cycle, especially the G1 to S-phase transition, the precise mechanism of action has not yet been explained. Bcl-2 overexpression has been implicated in the rescue of cells targeted for death by numerous stimuli, including GCs [30]. So, it would investigate whether RU486-mediated rescue of B cells was associated with the alternation of bcl-2.

The SNS, a major component of the ANS, innervates all lymphoid organs [31]. Stimulation of sympathetic nerves releases granules containing neurotransmitters including norepinephrine (NE) and catecholamines (CAs), which modulate immune response by multi-pathway [20]. NE is the major neurotransmitter released from sympathetic nerves and its receptors is β-adrenoreceptors expressed mostly on immune cells. In fact, NE released locally in lymphoid organs was recently reported to directly accelerate HIV-1 replication by up to 11-fold in acutely infected human PBMC [32]. Recently, a study report showed SNS increased proinflammatory cytokines and decreased the anti-influenza virus CD8 T response, and exacerbated influenza virus pathogenesis [9]. Splenic content of NE can be depleted up to 95% by the noradrenergic neurotoxin 6-OHDA [31]. In this study, the mice treated with 6-OHDA displayed an increased in the spleen B cells, BM early B cells and CD4+CD8+ thymocytes (data not shown) within H9N2 virus infection. So NE may be associated with the spleen B cells homeostasis and thymus T cells development.

Both HPA axis and SNS response blockade did not result in an overlapping effect comparing with individual blockade. The treatment did not fully recover spleen and BM B cells to the level of wild mice. Other than HPA axis and SNS response, there should be other factors responding for V virus infection induced depletion of B cells. So, it is necessary to investigate the molecular mechanism of loss of B cells after infection.

The brainstem receives inputs that signal major homeostatic perturbations, such as blood loss, respiratory distress, visceral or somatic pain and inflammation [20]. Signals of homeostatic imbalance in the brainstem lead to activation of the HPA axis and sympathetic responses to these inputs [20]. The responsiveness of SNS and the HPA axis plays an importan role in immune regulation and for the outcome of infection and inflammatory disorder. When homeostasis is disturbed or threatened by internal or external challenges resulting in increased peripheral levels of CAs and GCs that act in concert to keep the steady state of the internal milieu. Any immune challenge that threatens the stability of the internal milieu can be regarded as a stressor; i.e., under certain conditions an immune response can activate the stress system. In fact, TNF-a, IL-1, and IL-6 activate both the SNS and the HPA axis [33], [34]. However, another study revealed that the increased level of serum GCs induced by influenza virus infection was in part independent of a systemic inflammatory response [35]. The influenza virus infection working as stressor-related information was conveyed to the brain and resulted in the activation of HPA axis. Whether the V infection resulting in the higher GCs level in plasma was induced by stronger inflammatory response at early time of infection needed to be investigated further. In conclusion, this study provides an evidence for the role of the HPA axis and sympathetic responses in the depletion of spleen B cells and BM early B cells induced by H9N2 AIV.
